# Necessity, Norm and Missing Knowledge: What Modals Tell Us About Crisis Response in German COVID-19 Reporting

**DOI:** 10.1007/s41244-021-00212-4

**Published:** 2021-08-05

**Authors:** Marcus Müller

**Affiliations:** Institut für Sprach- und Literaturwissenschaft, TU Darmstadt, Darmstadt, Germany

**Keywords:** Modal Verbs, Crisis Communication, COVID-19 Discourse, Corpus Linguistics, Modalverben, Krisenkommunikation, COVID-19 -Diskurs, Korpuslinguistik

## Abstract

This study examines modal verbs in German press coverage of COVID-19 during the first phase of the pandemic. The data basis is an 18-million-word corpus of newspaper articles. For analysis, a sample is drawn from the total number of modal verbs in the corpus and these are categorised according to their discourse function. The corresponding annotated data are analysed quantitatively and qualitatively. For this purpose, the study draws back to Kratzer’s concept of conversational backgrounds. It turns out that in addition to normative speech backgrounds, goal formulations can be found above all. Normative backgrounds are evoked, on the one hand, to address official rules and their effects and, on the other hand in appeals and demands, to refer to social norms that are assumed as common ground. The fact that teleological backgrounds play a relatively large role indicates that the normalisation perspective is of great importance as a regulative in the crisis discourse. More positive than negative determining factors are indicated and uncertainty markings occur comparatively rarely. This points to successful crisis communication in this discourse phase.

## Abstract (de)

Diese Studie untersucht Modalverben in der deutschen Presseberichterstattung über die erste Phase der COVID-19-Pandemie. Die Datengrundlage ist ein 18 Millionen Wörter umfassendes Korpus von Zeitungsartikeln. Für die Analyse wird eine Stichprobe aus der Gesamtzahl der Modalverben im Korpus gezogen und diese werden nach ihrer Diskursfunktion kategorisiert. Die entsprechend annotierten Daten werden quantitativ und qualitativ ausgewertet. Dazu wird Kratzers Konzept der Redehintergründe verwendet. Es zeigt sich, dass neben normativen Redehintergründen vor allem Zielformulierungen zu finden sind. Normative Hintergründe werden aufgerufen, um einerseits behördliche Regeln und deren Wirkungen zu thematisieren und andererseits in Appellen und Forderungen auf soziale Normen zu verweisen, die als Common Ground vorausgesetzt werden. Dass teleologische Hintergründe eine verhältnismäßig große Rolle spielen, weist darauf hin, dass die Normalisierungsperspektive als Regulativ im Krisendiskurs eine große Bedeutung hat. Für eine erfolgreiche Krisenkommunikation in dieser Diskursphase spricht, dass mehr positive als negative Rahmenbedingungen angegeben werden und Unsicherheitsmarkierungen vergleichsweise selten auftreten.

## Introduction

The COVID-19 pandemic is widely perceived as one of the greatest crises since World War 2. It is a recognized fact in risk research that communication plays a central role in managing crises (WHO 2017). This is all the more true in the case of a viral pandemic, the spread of which depends on nothing but the number and type of social contacts. In such a situation, the actors in politics and administration are reliant on the population’s trust and willingness to cooperate. This article highlights a specific linguistic aspect of crisis communication: the use of modal verbs in public news discourse. This is because modal verbs play a key role in discourse when uncertainties, norms and expectations have to be dealt with linguistically. Specifically, it reports on a study on modal verbs in German press coverage from 20 January to 31 May 2020. Therefore, I draw on an 18-million-word corpus of newspaper articles. Because it is not possible to reliably determine the discourse functions of modal verbs automatically, this study is based on a sample that was categorised and evaluated manually.

To start by giving an idea of the importance of modal verbs in the COVID-19 discourse, I quote here parts of the first speech of the German Federal Chancellor Angela Merkel which she delivered directly to the population on the occasion of the COVID 19 pandemic. It was a television address on 18 March 2020^1^. In my discussion of the speech excerpts, I focus on constructions with modal verbs. Modal verb constructions are underlined. I describe their function non-terminologically at this point. Later, I will introduce the appropriate terminological distinctions:

[…] Millions of you can’t go to work, your children can’t go to school or daycare, […]. I turn […] to you because I want to tell you what guides me […] in this situation. […] I want to explain to you where we currently stand in the epidemic, […]. But I also want to convey to you why it needs you to do this, and what each and every individual can contribute. […], so that research can develop a drug and a vaccine. […] so that those who fall ill can receive the best possible care. […] Germany has an excellent healthcare system, […]. This can give us confidence. […] and at the same time we must focus on one thing, which is existential: to reduce public life as much as possible. […] and we want to preserve as much economic activity as possible. […] But everything that could endanger people, everything that could harm the individual, but also the community, we must reduce that now. We must limit the risk of one infecting the other as much as we possibly can. […] [Restrictions] should never be decided lightly and only temporarily in a democracy. […] The federal government is doing everything it can to cushion the economic impact […]. We can and will use everything it takes to help our entrepreneurs and employees through this difficult ordeal. […] And everyone can be assured that food supplies will be available at all times, […]. Just as indiscriminately any of us can be affected by the virus, so now everyone and anyone must help. […] This is what an epidemic shows us: […] how we can protect and strengthen each other by acting together. […] […] we must keep our distance from each other out of consideration. […] And really everyone must understand that: […] The well-intentioned visit, the trip that didn’t have to be, all of that can mean contagion and really shouldn’t happen now. […] the experts say: grandparents and grandchildren should not get together now. […] Whoever avoids unnecessary encounters helps everyone who must take care of more cases every day in the hospitals. […] We all need to find ways to show affection and friendship: […] We now hear of wonderful examples of neighbourly help for the elderly who cannot go shopping themselves. […] This is a dynamic situation and we will remain adaptive in it, to be able to rethink at any time and respond with other tools. […] We can respond now, decisively, all together. We can accept the current constraints and stand by each other. […] We must show, even if we have never experienced something like this before, that we act cordially and reasonably and thus save lives. […]

The first sentence cited here is the third sentence in the original speech. Merkel uses the negated construction *nicht können* (can’t) twice here to refer to external restrictions on action to which the population is subject. She forms the next three modal verb constructions with the modal verb *wollen* (want to). In each of these she embeds communication verbs, namely *sagen* (tell), *erklären* (explain), and *vermitteln* (convey). Thus, the modal verb constructions here indicate performative speech acts, they mark intentionality and have the function of establishing the axis of relation between chancellor Merkel and the television audience. This effect is reinforced by the climax of urgency inherent in the choice of communication verbs. This is followed by a series of constructions with *können* (can), all of which indicate positive possibilities for action, each with slightly different nuances. Merkel then uses constructions with *müssen* (must), *wollen* (want to) and the subjunctive forms of *können* (could) and *sollen* (should). These introduce a reference to the future and refer to necessities, goals, and risks. These modal backgrounds are intertwined by the alternating modal verbs in the text flow. This rather negative passage is in turn counterbalanced by *können* constructions, which Merkel uses to address capabilities of the population. The last modal verb of the speech is *müssen*: »We must […] show that we act cordially and reasonably and thus save lives.« This sentence is remarkable in two respects: First, it introduces an ambivalent action background with the verb *müssen*, which can refer to an external constraint as well as to a social norm. Both interpretive contexts were established earlier in the speech. With the use of *müssen* at the end of her speech (it is the third to last sentence) Merkel merges these two contexts. Second, the action verb *zeigen* (show), with which Merkel invites the television audience not only to act, but to manifestly perform the action, is embedded in this doubly charged context of action. She thus appeals to the sociality of actions in this situation of crisis. She reinforces this by using the adjective *herzlich* (cordially), which is extremely unusual for a *müssen* construction, and which incorporates the predication *Leben retten* (save lives). Thus, we can see that the modal verbs form the normative framework of the speech, as it were, with which Merkel establishes necessities, desires, external and internal constraints, possibilities and abilities as the background of her concrete appeal for action in a rhetorically strictly composed manner.

Merkels speech has been discussed as an extremely successful example of crisis communication. An expert jury at the Seminar for General Rhetoric at the University of Tübingen voted it the Speech of the Year 2020^2^. This insight into her use of modals may give a hint to why this is. The analysis above shows that it is precisely the balanced sequence of modal verbs that Merkel uses for the linguistic management of social uncertainty and normativity: she uses them to mark empathy, to establish the communication axis, to merge factual necessity and social norms, and thus leaves no doubt about the urgency of her appeal. But of course, this speech has been neither the only communication about actions and their conditions during the first phase of the corona pandemic nor can we say what concrete influence Merkel’s personal communication had on the course of the COVID-19 pandemic in Germany. It is therefore necessary to widen the focus and take a broader look at the public discourse of this period. For this purpose, I draw on a corpus of newspaper articles, since on the one hand they document public debates and policy statements, and on the other hand newspapers – despite the enormous spread and impact of social media – can still be regarded as leading media (Zinn 2020, p. 9 f.).

In chapter 2 I firstly introduce work on crisis communication and secondly, I provide terminological distinctions of the functional categories of modal verbs. In chapter 3 the corpus and methodology are introduced. I describe my sampling strategy and data categorisation. Chapter 4 provides the results of my study.

## Risk communication and modal verbs in Covid-19

### Crisis communication

The COVID-19 pandemic has influenced social life like hardly any other event since the founding of the Federal Republic of Germany. This existential crisis poses extreme challenges for the social actors who have to conduct or organise crisis communication. While communication is fundamental in any crisis, it is the decisive factor in a virus pandemic, whose spread depends solely on the social behaviour of the population. Social science contributions to crisis communication have highlighted that for the responsible actors in crisis communication, it is a matter of getting their addressees to act appropriately in as targeted a manner as possible. Older approaches model a strong knowledge gap between communicators and their addressees, i.e., they assume that the communicating institution knows exactly what is good for the population and, accordingly, it is only a matter of using simple and direct messages and information to persuade the addressees to take the right action (Lundgren/McMakin 2018, p. 24). In contrast, more recent approaches emphasise the complexity of risk discourses and the need to communicate reasons to citizens in addition to information for action: »Our own experience and a growing body of evidence suggest that people are more likely to change behaviour when they know the ›why‹, not just the ›what‹ or ›how‹« (Lundgren/McMakin 2018, p. 24). The Relational Dialectics Approach (Littlefield/Sellnow 2015) assumes the complexity of the communication situation that arises when a modern society is in crisis. It is not so easy to discern who is the subject and who is the addressee of communication at all, because all social actors can become relevant communicators. While communication through and with classic actors of state institutions, civil society, private organisations and media is already complex enough, the dynamics of social media fully turn the situation into a complex system in which monocausal connections can no longer be initiated. »The implication for risk communicators is to not only understand the audience but to involve the audience fully in crisis communication. This can be best accomplished during preparedness planning, but social media has also made it possible to work collaboratively with community members during a crisis« (Lundgren/McMakin 2018, p. 25).

Accordingly, crisis communication must not be imagined as a one-way activity, but as a polyphonic social process (Fischhoff 1998). It should be noted that risks in crisis scenarios are not purely objective facts, but are always imbued with value concepts (Renn 2008). In the COVID-19 pandemic, after an initial phase in which the various social groups were largely able to agree on the goal of massively reducing social contacts in order to ensure the protection of the lives of vulnerable groups as much as possible, more and more discourse agents introduced complementary perspectives. In doing so, they have pointed not only to economic dislocations for individuals and society, but also to the massive psychosocial and somatic impairments of entire social groups.

Crisis communication in modern societies must therefore not only deal with a central risk, but also with different interests, values and relevance markings (Thompson 2008). Moreover, crisis communication is confronted with emotions (Lupton 2013) as well as fears and hopes (Zinn 2016), which must be verbalised and addressed, but not functionalised or disappointed. Müller, Bartsch and Zinn (in press) have investigated the verbalisation of uncertainty in the first phase of the pandemic in Germany and the UK and found, among other things, that the marking of disagreement in Germany increases close to a first phase of high agreement, while disagreement in the UK remains at about the same level over the first 4 months of the pandemic. They point out that the relationship between social uncertainty and its linguistic representation is complex:

Successful leadership in crisis situations demands unambiguous communication of the situation and necessary responses. At the same time, research shows the necessity to communicate uncertainties in a way that does not jeopardise trust in decision makers, and to secure compliance (Covello 2009). As Jaspal & Nerlich (2021) have argued, the ambiguous language the British Prime Minister Boris Johnson used to communicate social distancing measures led to confusion and inefficient responses. Thus, uncertainty situations require unambiguous language rather than markers of uncertainty (Müller/Bartsch/Zinn in press)

It appears from this short research report that crisis communication is concerned with the ambivalence of uncertainty and clarity, confusion and control. We have seen above from Angela Merkel’s speech what an important role modal verbs can play in dealing with this. In fact, modal verbs are overrepresented in the corpus of newspaper articles on COVID-19 (cf. 3.2). After presenting the basics for the linguistic description of modal verbs, I will show which role the modal verbs play in detail.

### Modal verbs

As we have seen discussing Angela Merkel’s speech, modal verbs can make an important contribution to the linguistic management of the complex discourse formation in crisis situations. This has mainly to do with the fact that – as already shown above – they introduce backgrounds such as norms, assumptions, abilities, goals or constraints into the discourse and link them to the thematisation of actions. Kratzer calls those modal contexts »conversational backgrounds« (Kratzer 1981, 1991). These are in part highly context-sensitive and in part bound to certain syntactic constructions (Boogaart/Fortuin 2016; Müller/Stegmeier 2019, pp. 328–331). Modal verbs in German are *müssen* (must), *sollen* (should), *dürfen* (may/be allowed), *können* (can), *mögen* (want to), *wollen* (want to).^3^ All of these verbs are highly ambiguous. Zifonun, Hoffmann and Strecker (1997, p. 1884) use the concept of conversational background to differentiate functional types of modal verbs: »By a (single) conversational background we mean the preconditions in view of which the pending draft of facts is to be evaluated (as possible or necessary).«^4^ They distinguish the following types of conversational backgrounds. The illustrating examples are taken from Angela Merkel’s speech cited above, if they occurred there. Other examples are given with references.

Epistemic background

The modal connects the speaker’s own perception and its level of certainty to the embedded proposition.

*Aber alles, was Menschen* *gefährden* *könnte, alles, was dem Einzelnen, aber auch der Gemeinschaft* *schaden könnte, das müssen wir jetzt reduzieren.* [But everything that could endanger people, everything that could harm the individual, but also the community, we must reduce that now.]

Normative background

The modal connects the embedded proposition to norms or values.

*Wir* *müssen das Risiko, dass der eine den anderen ansteckt, so* *begrenzen, wie wir nur können.* [We must limit the risk of one infecting the other as much as we can.]

Teleological background

The modal verb relates the embedded proposition to an objective in the future.

*[…] wir* *wollen so viel wirtschaftliche Tätigkeit wie möglich* *bewahren.* [[…] we want to preserve as much economic activity as possible.]

Volitive background

The modal verb constitutes the embedded proposition as an expression of will or preference of the uttering person.

*Ohne Mund-Nasen-Maske darf der Drogeriemarkt im Cannstatter Bahnhof nicht betreten werden. […] Doch ein 43-Jähriger hat keinen Mundschutz dabei, und er* *will das nicht* *akzeptieren.* (Stuttgarter Zeitung, 15.05.2020) [People are not allowed to enter the drugstore at Cannstatt station without a mouth-nose mask. […] But a 43-year-old man does not have a mouth mask with him, and he does not want to accept that.]

Circumstancial background

(Zifonun/Hoffmann/Strecker 1997, p. 1884) distinguish between intrasubjective and extrasubjective circumstantial backgrounds. This refers to internal or external circumstances of the realisation of an action, for example, whether someone can achieve something through his or her own strength or competence (intrasubjective) or is prevented from doing so by external circumstances (extrasubjective). In our context, it is above all important whether the circumstances promote the realisation of action or whether they hinder it. Therefore, I introduce the distinction between circumstantial-restrictive backgrounds and circumstantial-enabling backgrounds:

Circumstancial-restrictive background

The modal connects the embedded proposition to restricting conditions.

*[…] Millionen von Ihnen* *können* *nicht zur Arbeit, Ihre Kinder* *können nicht* *zur Schule oder in die Kita, […].* [[…] Millions of you cannot go to work, your children cannot go to school or nursery, […]]

Circumstancial-enabling background

The modal connects the embedded proposition to enabling conditions.

*Die Bundesregierung tut alles, was sie* *kann, um die wirtschaftlichen Auswirkungen abzufedern […] Wir* *können und werden alles* *einsetzen, was es braucht, um unseren Unternehmern und Arbeitnehmern durch diese schwere Prüfung zu helfen.* [The Federal Government is doing everything it can to soften the economic impact […] We can and will do everything we it takes to help our employers and workers through this difficult test.]

In addition, I introduce the following conversational backgrounds, which play an important role in Angela Merkel’s speech and are not covered or specified by the account in Zifonun, Hoffmann and Strecker (1997, p. 1882):

Potential background

I use the term potential background to refer to cases in which possible facts in the past, present and future are spoken about non-epistemically as objective possibilities. Zifonun, Hoffmann and Strecker (1997, p. 1885 f.) include these cases under the extrasubjective circumstantial conversational background. In our context, however, it is important to distinguish between action-enabling and action-restricting circumstances on the one hand and logical or objective-empirical possibilities of an action or process occurring on the other, as these each have a different activation potential in crisis communication. In philosophy and semantics, this is referred to by the term ›alethic modality‹.^5^

*So wie unterschiedslos jeder von uns von dem Virus* *betroffen sein* *kann, so muss jetzt auch jede und jeder helfen.* [Just as indiscriminately any of us can be affected by the virus, so now everyone and anyone must help.]

Rhetorical background

The rhetorical background describes the speaker’s intention by marking an explicitly performative speech act in order to establish an axis of relation between the speaker and the listener(s).

*Ich wende mich […] an Sie, weil ich Ihnen* *sagen will, was mich […] in dieser Situation leitet.* [I turn to you […] because I want to tell you what guides me […] in this situation.]

It is important to emphasise at this point that modal verbs are systematically ambiguous, i.e. there is no linear assignment of modal verbs to conversational backgrounds.* Können* for example may receive epistemic as well as circumstancial interpretations (Zifonun/Hoffmann/Strecker 1997, p. 1885). Moreover, individual conversational backgrounds can be indexed by several modal verbs. For instance, the normative speech background can be indexed by the modals *müssen, sollen, können* and *dürfen*. Therefore, measuring modal verbs in a corpus can only be a first step. For functional determination, one has to disambiguate them.

## Corpus and methodology

### Corpus

**Fig. 1:**
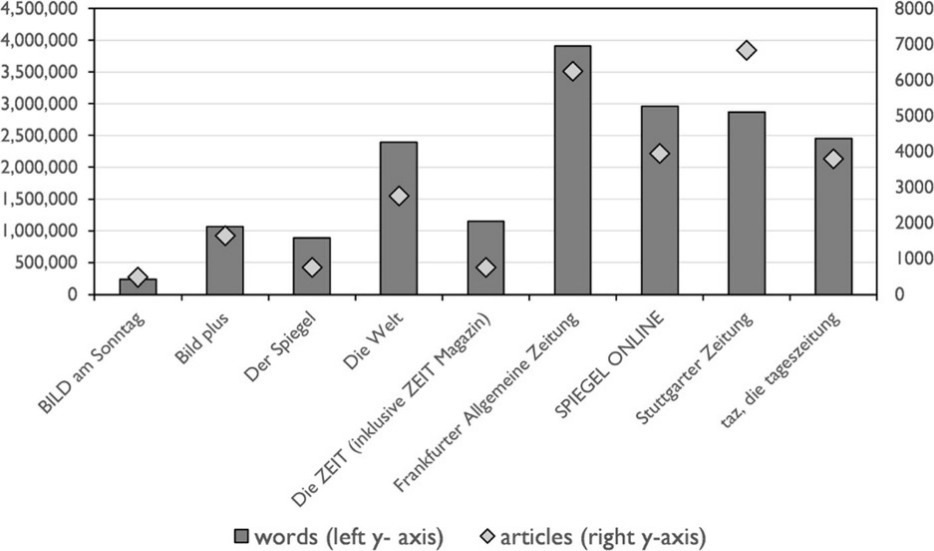
Corpus

The data used in this study consists of articles on the coronavirus pandemic from 20 January to 31 May 2020.^6^ They have been retrieved from the media databases LexisNexis and ProQuest using the query terms: corona*, sars-cov‑2 and *covid-19.* The corpus comprises 27,136 articles and 17,918,177 words (tokens) from the following newspapers: BILD am Sonntag, Bild plus, Der Spiegel, Die Welt, Die ZEIT (comprising ZEIT Magazin), SPIEGEL ONLINE, Stuttgarter Zeitung, taz. The corpus texts were tokenised, lemmatised and POS-tagged^7^. POS-tagging has been carried out with spaCy^8^ using the Stuttgart-Tübingen-Tagset (Schiller et al. 1999). Sentences were segmented. All manual annotation and measurements reported in this article have been carried out in CQPWeb (Hardie 2012). In Figure 1 the ratio between number of words and number of articles for each newspaper is displayed.

**Fig. 2:**
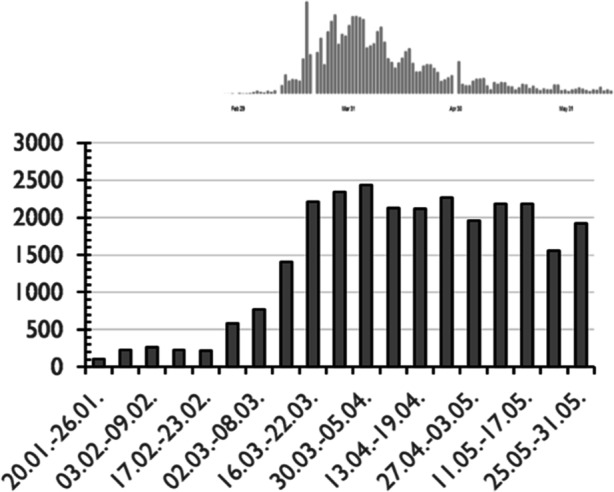
No. of articles per week – above: new SARS Cov‑2 infections in Germany

The articles are distributed over the calendar weeks as displayed in Figure 2. Not surprisingly, there is a connection between the number of new infections and the number of articles per week. The increase in articles starts about a week before the increase in new infections in the first wave. While these subside again at the beginning of April, the number of published press articles remains at a constant level because the restrictions for the population and the need for negotiating political measures remained.

### Measurements, sampling and categorisation

I first measured all modal verbs as listed above in this corpus. In order to assess the importance of modal verbs for the COVID-19 discourse, two questions need to be answered first: Firstly, are modal verbs more frequent in the corpus than can be expected? And secondly, does the frequency of modal verbs change over the study period? To answer the first question, I compared the frequency of the modals in the COVID-19 corpus with their frequency in a reference corpus (Scott 2009). I used a dataset of randomly selected newspaper articles from 2013–2017. I compared not only the frequencies of modal verb occurrences but also the percentage of negated modal verb constructions.^9^ This is because modal verbs are systematically related to one another in the system and negations play a major role in this. For example, *nicht dürfen* is the negative variant of *müssen* in indexing the normative background. I addressed the second question by measuring the distribution of modal verbs across calendar weeks. To enable such measurements, ›calendar week‹ was created as a structural XML attribute in the text model of the corpus.

In order to estimate the discourse-functional role of modal verbs in the COVID-19 pandemic, I drew a random sample. The total number of modal verbs (tokens) in the corpus is 228,669, of which I randomly selected 1%, i.e. 2,287 occurrences of the modal verbs listed above.

**Fig. 3:**
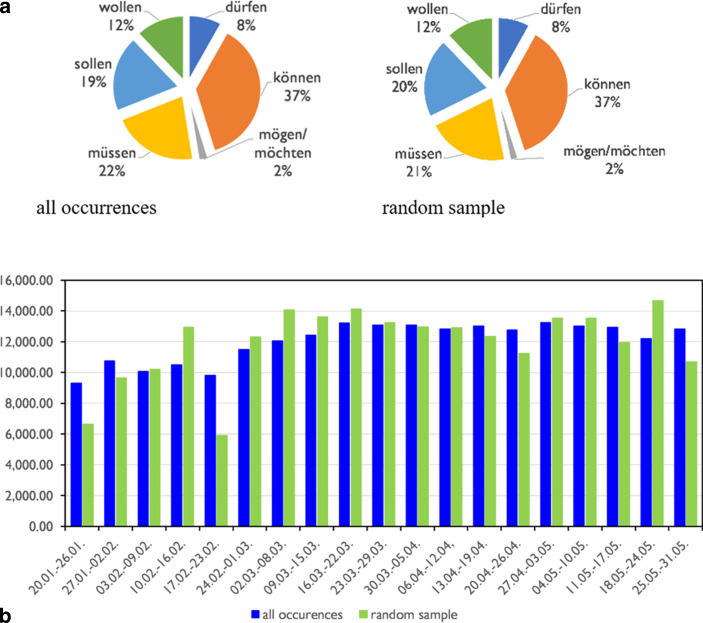
**a** Distribution of modals in the full data and in the random sample. **b** Distribution of modals in the full data and in the random sample across weeks

The distribution of modal verbs in the sample is shown in Figure 3a (right). In order to assess the representativeness of the sample, I compared it with the distribution of modal verbs in the population as a whole (Fig. 3a, left). One can see that there is only a difference of 1 percentage point for *sollen* and *müssen*. When comparing the distribution of modal verbs in the sample and the population by calendar week (Fig. 3b), it can be seen that there is a significant deviation in the proportion of modal verbs between the sample and the population only in three weeks, the first, the third and the fourth of the measurement period. Since the data basis in the first week is still very small (cf. Fig. 2), this is not surprising, but indicates that the sample is sufficiently large and representative. However, the deviations in the first weeks must be considered when interpreting the results.

After measuring the development of the modal verbs across the study period, I categorised the modal verbs in the sample according to conversational backgrounds. I analysed the categorised data quantitatively. Afterwards, I collected the most important usage patterns of the modal verbs – structured according to conversational background – and analysed the discourse function of these patterns using concordances and sample evidence as examples.

## Results

### Form: POS-Distribution

**Fig. 4:**
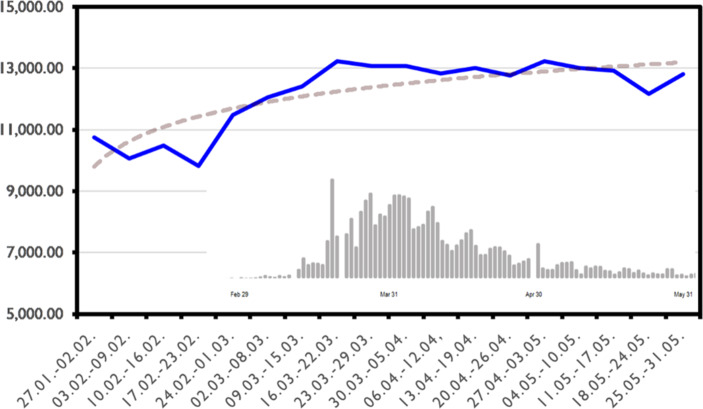
Development of modals in COVID-19 discourse and no. of new infections, f/1 Mill. words

A first observation of the quantitative development of all modal verbs over time shows that there is a shifted correlation between this and the development of infection numbers (Fig. 4). The relative frequency of the word type ›modal verb‹ was measured. Like the absolute number of articles, the relative frequency of modal verbs within the articles increases abruptly: In the week from 24.02. to 31.03. we measure a 17% increase in the frequency of modal verbs. This is remarkable in so far as modal verbs are frequently used in language anyway and the increase cannot simply be explained by thematic context or genre. In fact, this is the week in which Shrove Monday and Tuesday fall and the week in which two first major outbreaks were reported in the Heinsberg and Göppingen districts, both related to Shrove celebrations. This connection indicates that the outbreak of the COVID-19 pathogen in this week’s reporting is finally changing from an international media event to a national crisis with implications for action. Like the absolute number of articles, the proportion of modal verbs remains at a high level and does not decrease as the number of cases drops.

**Fig. 5:**
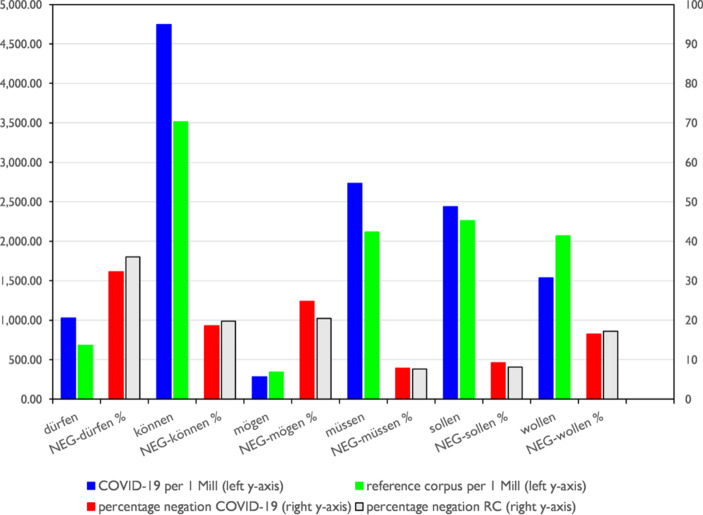
Modals in COVID-19 print media discourse – left y‑axis: relative frequency of modal verbs, right y‑axis: percentage of negated modal verb constructions

Figure 5 shows the quantitative relationship of the modal verbs to each other and to the frequency of the corresponding verbs in the reference corpus. The left y‑axis shows the relative frequency of the individual modal verbs, the right y‑axis the respective percentage of negated modal verb constructions. First of all, it shows that the proportion of the respective modals in the total system of modal verbs does not differ significantly compared to the reference corpus – with two exceptions: *Müssen* is proportionally more frequent and *wollen* is significantly less frequent. If we compare the relative frequencies of the modal verbs in relation to the corpus and reference corpus, we notice that the difference is greatest for *können*. In a keyword analysis (Gabrielatos 2018) that ranks according to the Log-likelihood score, *können* comes at rank 20 with Log-likelihood ratio (LLR) = 1564.91, which is quite surprising in a thematic corpus. It is thus in the list before words like *hospital, relaxation* or *infect*.^10^

*Müssen* is also highly significantly more frequent than in the reference corpus (rank 111, LLR 650.72). This also applies to *dürfen* (rank 127, LLR 598.73), which, however, occurs much less frequently overall. In the case of the frequent modal *sollen* the distance between the frequency in corpus and reference corpus is lower, but still highly significant (rank 1634, LLR 56.77). *Wollen* and – much rarer in evidence – *mögen* occur significantly less often in the corpus than in the reference corpus. The proportions of negated modal verb constructions do not differ significantly from those in the reference corpus. The construction *nicht können*, which is prominently used in Angela Merkel’s speech, only has a share of approx. 19% of the *können* occurrences and is thus no more frequent than in the reference corpus. This may be surprising, since the COVID-19 discourse necessarily refers to situational restrictions, which are typically indicated by *nicht können*.

### Function: Conversational backgrounds in the sample

#### Distribution

**Fig. 6:**
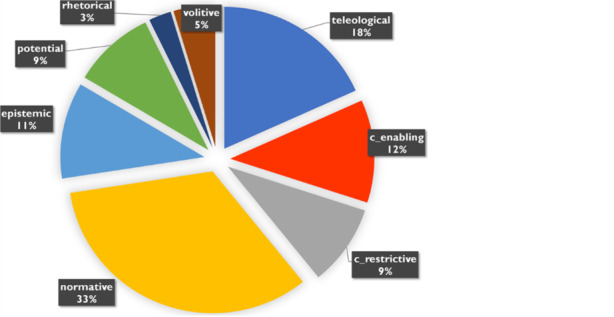
Conversational backgrounds in the sample

The 2,287 modal verb constructions in the sample were now manually categorised according to the types of conversational backgrounds introduced above (Fig. 6). 33% percent of the occurrences indicate the normative conversational background, 18% the teleological and a total of 21% the circumstantial speech background, of which slightly more the enabling and slightly less the restrictive circumstantial background. The epistemic conversational background is used in 11% of the cases, while the volitive background is negligible at 5%. Although the rhetorical background took on an important function in Angela Merkel’s speech, it does not carry any quantitative weight in the press corpus here with 3%.

Two aspects of this distribution may be surprising: Firstly, the addressing of restrictions on action with modal verb constructions is rarer than might have been expected in reporting on a pandemic that has to be fought through restrictions on freedom of movement and action. And secondly, the share of epistemic speech backgrounds is also relatively small, considering the high degree of uncertainty with which the discourse was necessarily associated. However, in addition to epistemic speech backgrounds, we find an almost equal share of non-epistemic potential backgrounds. This means that possibilities and risks are rendered about equally often with and without a component of subjectification. In such an uncertain situation as the first phase of the COVID-19 pandemic, it is still remarkable that target formulations introduced with the teleological background account for just under one fifth of the evidence in the sample.

#### Patterns

This section provides a detailed analysis of those conversational backgrounds that have proven to be quantitatively discourse-relevant: the normative, the circumstantial, the teleological as well as the epistemic and potential. In doing so, I will show which modal verbs are used to index the conversational backgrounds in discourse, work out patterns of indexing and give discourse-functional descriptions on the basis of textual evidence.

##### Normative background

**Fig. 7:**
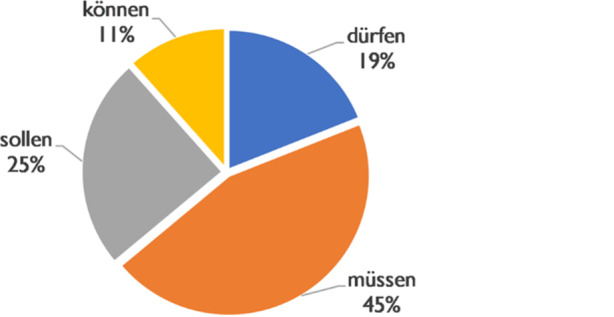
Modals and normative conversational background

The normative background is indexed in the corpus by four modal verbs (Fig. 7). The most frequent is *müssen* (45%), *sollen* is used in 25% of the cases, *dürfen* in 19% and *können* in 11%. Negated constructions are included. The variation indicates the range of references to norms, values and socially effective expectations. The various modal verbs each bring central aspects of meaning into the norm reference, which are specified in use firstly by the semantics of the embedded proposition and secondly by contextualisation. In the case of *müssen*, this is ›compulsion‹, in the case of *sollen* ›demand‹, in the case of *dürfen* ›permission‹ and in the case of *können* ›possibility‹. While *müssen, sollen* and *dürfen* have their domain in the normative background, in the case of *können* the communicative potential of the circumstantial background is introduced. Normative thematisations with *(nicht) können* are thus conceptualised with an overtone of no alternative.

We find *müssen* essentially with two functions in the data: Firstly, as a reference to state or official rules and regulations (a) and secondly, in demands of individuals and groups, in which the speakers – often implicitly – back up the urgency of their demand by referring normatively to the common ground (b).

- *Kommen Auflagen, dass man* *nachweisen muss, nicht infiziert zu sein, einen Impfschutz oder eine anderweitige Immunität zu haben?* SPIEGEL online, 11.05.2020 [Will requirements be introduced that one must prove not to be infected, to have vaccination protection or other immunity?]
- *Mit der Digitalisierung* *muss es das Ziel sein, dass Praxen und Kliniken künftig nicht nur in der Krankenversorgung, sondern auch in der Forschung viel enger als bisher zusammenarbeiten.* Bild plus, 21.04.2020 [With digitalisation, the goal must be for practices and clinics to work together much more closely in future than they have in the past, not only in patient care but also in research.]

*Sollen* occurs in the same contexts as *müssen*, except that it formulates official regulations as more or less urgent recommendations (c). In modal verb constructions that indicate demands, *sollen* also weakens the claim to validity of the asserted proposition (d).

c) *Man solle öffentliche Verkehrsmittel* *meiden, empfahl die Bundeskanzlerin, also bestellte ich mir ein Taxi.* taz, 16.03.2020 [One should avoid public transport, the Chancellor recommended, so I ordered a taxi.]
d) *Nachdem auch Fitnessstudios und Spielhallen wieder öffnen dürfen,* *sollten unsere Kinder langsam mal* *dran sein*. Die Welt, 22.05.2020 [Now that gyms and gambling halls are allowed to open again, it should be our children’s turn.]

In the use of the modal verb *dürfen* we also find the dichotomy between state and official regulations (e) and presupposed common ground (f) as a normative background. Both the positive normative reference (permission) and the negative one (prohibition or negative demand) mark an absolute claim to validity of the proposition.

e) *Den Spielplatz* *darf nur* *betreten, wer keine Symptome hat, die auf Covid-19 hindeuten.* Bild plus, 03.05.2020 [Only those who do not have symptoms that indicate Covid-19 are allowed to enter the playground.]
f) *Was nicht* *passieren darf: Dass die EU-Chefs wieder mal einen lauwarmen Kompromiss präsentieren.* SPIEGEL online, 23.04.2020 [What must not happen is that the EU leaders once again present a lukewarm compromise.]

We find *können* in comparable constructions, with the difference that the norm is conceptualised as an enabling or (in negated constructions) restricting circumstance of the modalised action (g). This has the effect that the agents who set the norms move into the background or disappear entirely. The norm is presented as inevitability, which on the one hand implies an absolute claim to validity and on the other suspends the power relations that always resonate in norm references.

g) *Eine kleine Trauergemeinde mit maximal zehn Personen* *kann* *sich während der Bestattung direkt am Grab oder der Urnenwand* *zusammenfinden*. Stuttgarter Zeitung, 27.03.2020 [A small group of no more than ten mourners can gather directly at the grave or urn wall during the funeral.]

An evaluation of the formulations with which the normative conversational background is introduced into discourse has revealed the following patterns^11^. In cases where patterns feature slots with certain semantic properties, I note them in the conceptual framework of frame semantics. I use the terminology developed in Frame-Net for this purpose.^12^ Formulations that occur 3 times or more often in the sample are included here:^13^

**CITIZEN**

MUSS / SOLL

*Abstand / s. an Regel halten* [keep distance / abide by rules]

*auf* NP_[Content]_ *verzichten* [renounce NP_[Content]_]

*es (eigentlich) wissen* [actually know sth.]

NP_[Message:allowance]_ *beantragen* [apply for NP_[Message:allowance]_]

NP_[Topic]_ *ernst / nicht auf die leichte Schulter nehmen* [take NP_[Topic]_ seriously / not take NP_[Topic]_ lightly]

*Handschuhe / Masken tragen* [wear gloves or masks]

*zuhause arbeiten* [work at home]

*sich / andere schützen* [protect oneself and others]

*Regeln einhalten* [respect rules]

*Steuern / Strafe zahlen* [pay taxes / penalty]

*zu Hause / auf dem Zimmer / im Krankenhaus bleiben* [stay at home / in the room / in the hospital]

DARF NICHT/KANN NICHT

NP_[Source]_ *verlassen* [leave NP_[Source]_]

DARF / KANN

NP_[Message]_* beantragen* [apply for NP_[Message]_]

NP_[Decision]_ *selbst entscheiden* [decide NP_[Decision]_ for oneself]

*im Büro / zuhause arbeiten* [work in the office at home]

*Unterstützung / Geld / Hilfen bekommen* [get support / money / help]

*zu Besuch bei* NP_[Goal]_ *kommen* [come to visit NP_[Goal]_]

*in den Genuss von* NP* kommen* [benefit from NP_[Benefactor]_]

*wieder Fußball spielen* [play football again]

**COMPANY / GASTRONOMY**

MUSS

*ab* UHRZEIT* / bis* DATUM* schließen* [close from TIME / until DATE]

*Steuern / Strafe zahlen* [pay taxes / penalty]

DARF

*(wieder) öffnen* [(re)open]

*(Veranstaltung) stattfinden lassen* [let an event take place]

DARF NICHT

*öffnen* [open]

**ADMINISTRATION / GOVERNMENT**

MUSS / SOLL

*alles tun, um* VP_INF_ [do everything to VP_INF_]

NP_[Goods]_* bezahlen* [pay for NP_[Goods]_]

NP_[Decision]_* entscheiden* [decide NP_[Decision]_]

NP_[Self_mover]_* in Gang / die richtigen Schwerpunkte / auf flexible Lösungen setzen* [get NP_[Self_mover]_ going / the right priorities / rely on flexible solutions]

*Unterstützung / Geld / Hilfen geben/gewähren/leisten* [give/grant/provide support / money / assistance]

It is noticeable that the negated norm-related constructions appear very sparsely in the list above. This is because they show a high variation in the sample and specific constructions occur less than 3 times. Overall, the negated modal verb constructions with a normative background represent about 15% of the cases in the sample. Examples are: ›s. must not take sth. lightly‹, ›s. must not take risks‹, ›s. must not meet s. with s.‹, ›must not praise the day before the evening‹, ›s. must not go to the playground‹.

##### Circumstancial-restrictive background

**Fig. 8:**
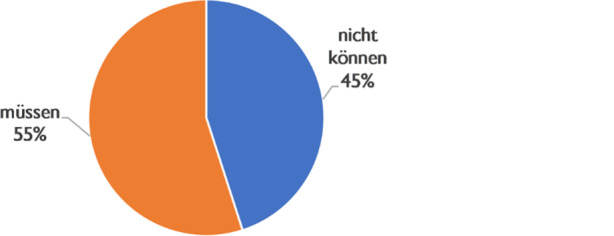
Modals and circumstantial-restrictive conversational background

The circumstantial-restrictive background is introduced in roughly equal parts by the modal verb constructions *müssen* (55%) and *nicht können* (45%) (Fig. 8). With *müssen*, the set of all potential possibilities for action is narrowed down to one that is mandatory or required by the circumstances. With *nicht können*, a presupposed positive possibility of action is excluded. As shown above, *müssen* has its domain in the normative background. In the evidence categorised here, the *müssen* constructions are used to thematise an unavoidable constraint caused by the situation. Many formulations are found in which the pressure to act with an absolute claim to validity is introduced by reference to a conversational background that exhibits a certain ambivalence between circumstance and norm. We have already seen the rhetorical function and the activation potential of such constructions in Angela Merkel’s speech.

The range of conversational backgrounds indexed by *must* can be seen in the evidence cited here. In the first case, the modal verb addresses the medical inevitability triggered by the virus (a). The second instance, on the other hand, represents a prompting action, in which an external necessity is referred to, which – actually or presumably – arises from the pandemic situation (b). What is exemplary about this citation is that the demand (*Wir müssen …*) and its backing (*um zügig …*) are connected on the basis of a topos with normative effect (cf. Wengeler 2015). It may be called the innovation topos: ›Whoever wants to be successful in solving a problem needs innovation and creativity.‹ Thus, in citation (b), the ›pure‹ extrasubjective circumstance as the context of the modalised action (as in a) becomes a norm-penetrated circumstance emulated under the conditions of society. Like almost all modal verbs with a normative reading, the construction has a teleological subsidiary meaning, because the call formulated here is aimed at the future. However, this is only triggered by the normative reference.

- *Er sagte, dass er sich verabschieden wolle, weil er für 7 bis 12 Tage* *ins künstliche Koma muss.* Bild plus, 26.04.2020 [He said that he wanted to say goodbye because he had to go into an artificial coma for 7 to 12 days.]
- *»Wir* *müssen unsere Innovation und Kreativität im Land schnell* *zusammenbringen, um zügig die beste Balance zwischen optimalem Gesundheitsschutz und rationalen datenbasierten Entscheidungsgrundlagen für das Anfahren von Wirtschaft, Bildung, Tourismus und Sport zu erlangen«, sagt Rolfs.* FAZ 12.05.2020 [»We need to quickly bring together our innovation and creativity in the country to quickly achieve the best balance between optimal health protection and rational data-based decision-making to drive the economy, education, tourism and sport«, says Rolfs.]

We find a similar spectrum of contextualisations in evidence with *nicht können*. Citation (c) represents a restriction on action due to external circumstances, while the negated modal verb construction in document (d) refers to an intrasubjective-circumstantial restriction with which an argument is made for the demand to accept the situation. The citation is taken from an interview with a psychologist. It can be seen that the modal verb construction occurs in the course of illustrating the concept of ›uncertainty‹ introduced earlier. The restriction introduced descriptively by the psychologist thus refers indirectly to the epistemic conversational speech background, which is introduced here on the second level.

c) *Dafür lieh er sich Geld, das er nun* *nicht zurückzahlen kann.* FAZ, 23.05.2020 [For this he borrowed money that he now cannot pay back.]
d) *Die Unsicherheit ist ein ständiger Begleiter in der Krise.* *Niemand kann abschätzen, was sich in diesen Wochen und Monaten verändert. Menschen müssen ihren Alltag in einer Geschwindigkeit neu strukturieren, die ihnen fremd ist. Die bekannte Ordnung erodiert. »Wir müssen diesen Zustand jetzt akzeptieren«, sagt Scheuermann.* Der Spiegel, 11.04.2020 [Uncertainty is a constant companion in the crisis. No one can estimate what will change in these weeks and months. People have to restructure their everyday lives at a speed that is alien to them. The familiar order is eroding. »We have to accept this state of affairs now«, says Scheuermann.]

From the patterns of modal verb constructions with indication of circumstantial restrictive backgrounds, we see that restrictions are highly related to the life of the individual citizen. We find verbs of inner feeling in the patterns (›experience‹, ›learn‹, ›come to terms with sth.‹), as well as those of outer activity (›work‹, ›help‹, ›do sth.‹). We see through these parallel pattern formations that intrasubjective and extrasubjective restrictions are brought close together in the discourse, so that the external situation and psychological constraints become blurred. Interestingly, we do not find any patterns here that indicate restrictions for states, institutions and actors in politics and administration.

**CITIZEN**

MUSS

*arbeiten* [work]

*eine Behandlung / Infusion bekommen* [get a treatment / infusion]

*für* NP_[Purpose]_ */ gegen* NP_[Side 2]_ *kämpfen* [fight for NP_[Purpose]_ / against NP_[Side 2]_]

*etw. erfahren* [find out about sth.]

NP_[Stimulus]_ *erleben* [experience NP_[Stimulus]_]

*mit* NP_[Theme:situation]_* leben* live with NP_[Theme:situation]_

NP_[Skill]_* lernen* [learn NP_[Skill]_]

*sich mit* NP_[Theme:situation]_ *abfinden* [come to terms with NP_[Theme:situation]_]

*sterben* [die]

KANN NICHT

*es sich leisten, zu* VP_INF[Activity]_ [*afford to* VP_INF[Activity]_]

NP_[Beneficiary]_ *helfen* [assist NP_[Beneficiary]_]

NP_[Counteragent:relatives]_ *sehen* [see NP_[Counteragent:relatives]_]

NP_[Activity:job]_ *ausüben* [pursue NP_[Activity:job]_]

##### Circumstancial-enabling background

**Fig. 9:**
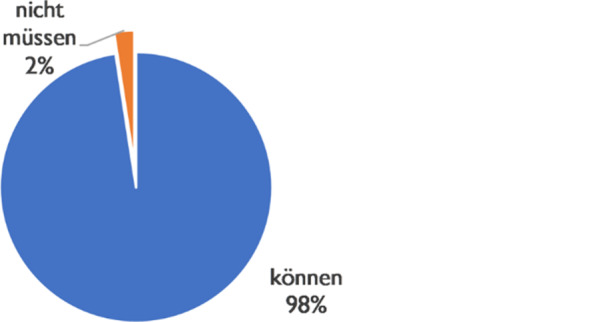
Modals and circumstancial-enabling conversational background

The circumstantial enabling background is almost exclusively indicated by the modal *können* (98%). The negating formulation by *nicht müssen* plays no role with only 2% of the evidence (Fig. 9).

The positive circumstantial backgrounds in the press discourse on COVID-19 can be located on three levels: Firstly, in factual asserted propositions about possible actions (a), which can be found according to the course of the epidemic in May 2020. Secondly, in non-factual embedded propositions that are subordinated to predicates of hoping, waiting and demanding (b). And thirdly, the corresponding modal constructions are found in embedded non-factual propositions, with matrix constructions referring on planning games and hypothetical models in crisis teams (c).

- *Damit* *können wir nun flächendeckend in die Sportvereine und aktive Bewegung* *zurückkehren.* FAZ, 07.05.2020 [This means that we can now now return to sports clubs and active movement across the board.]
- *Lola wartet darauf, dass der Spielplatz aufmacht und sie* *schaukeln* *kann*. taz, 27.04.2020 [Lola is waiting for the playground to open so she can swing.]
- *Einmütig verfassten sie Papiere, wie der Staat mit hohen Krediten einen Kollaps der Konjunktur* *verhindern kann*. Der Spiegel, 30. Mai 2020 [They unanimously wrote papers on how the state can prevent a collapse of the economy with large loans.]

According to this differentiated discourse semantics, the contexts of action to which positively enabling modal verb constructions are attributed are more diverse than in the case of restrictive-circumstantial constructions. The subjects of the corresponding modalised propositions can be citizens, but also events. A pattern is found in the sample related to government and administration.

**CITIZEN**

KANN

NP_[Stimulus]_* (deutlich) sehen* [see NP_[Stimulus]_ (clearly)]

*(noch) (gut) schlafen* [(still) sleep (well)]

*sich erinnern* [remember]

*(wieder) arbeiten* [work (again)]

*(eine bestimmte Zeit noch) durchhalten* [(still) hold out (for a certain time)]

*sich / einen Anspruch durchsetzen* [to assert oneself / a claim]

**MEASURE / EVENT**

KANN

*beginnen* [begin]

NP_[Beneficiary]_* vor Ansteckung / COVID-19 schützen* [protect NP_[Beneficiary]_ from infection / COVID-19].

*stattfinden* [take place]

**GOVERNMENT / ADMINISTRATION**

KANN

*den Zeitplan (unter Bedingung X) einhalten* [keep to the schedule (under condition X)]

**Fig. 10:**
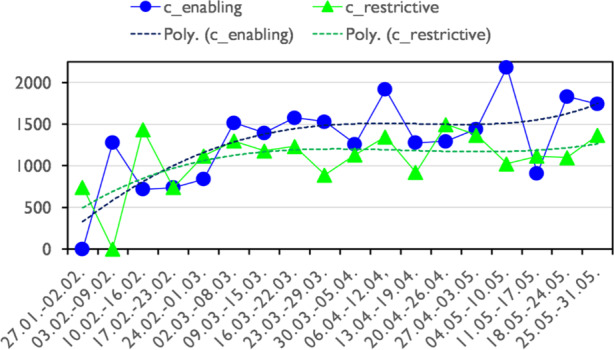
Circumstancial restrictive and circumstancial enabling background – development across weeks - f/1 Mill. (extrapolated from sample; Poly. = polynomial trendlines)

It is interesting to look at the quantitative distribution of restrictive and enabling circumstantial contexts by calendar week (Fig. 10). The graph should be viewed with caution because it shows data extrapolated from the sample in relation to the population and the sample is unreliable in the first weeks due to the small data basis. From March onwards, however, the data sample is representative, as shown above. The tendency of constructions with enabling backgrounds to become proportionally more frequent intensifies towards the second half of May, when enabling modal constructions are evidenced about 25% more often than restrictive modal constructions. This seems to be due to the pandemic development and the opening perspective after the first lockdown in May.

##### Teleological background

**Fig. 11:**
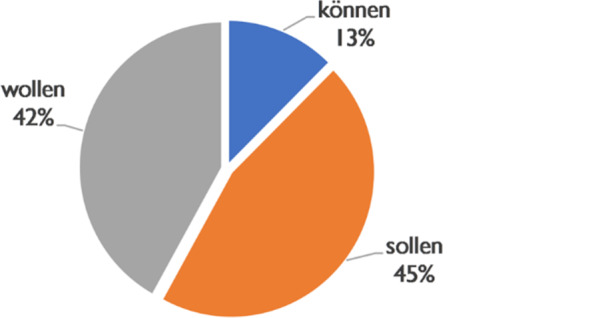
Modals and teleological conversational background

As we have seen above, the teleological background plays a considerable role in the discourse with 18% of the evidence from the sample. The introduction of objectives through modal verb constructions is performed prototypically through the non-psychological use of *wollen* (cf. a). This is true (see Fig. 11) in 42% of the cases. 45% are formed with the verb *sollen* (b). However, there are also 13% of cases with *können*, in which the teleological meaning is given a circumstantial-possible secondary meaning (c).

The majority of the evidenced teleological modal verb constructions (67% – 277 from 413) fall in the months of April and May, that is the phase of lockdown and relaxations. It can be noted that formulations of goals in April tend to be small-scale and oriented towards the next steps to take (as in a), while in May after the first wave of the pandemic perspectives are mostly directed towards medium-term (b) and long-term (c and d) goals. The citations (a) and (b) show that the choice between *wollen* and *sollen* as teleological markers is related to the marking of the agent of the goal formulation: While the agent is coded as a grammatical subject in *wollen* constructions (a), it is typically elided in *sollen* constructions (b). In *sollen* constructions, predicates with passive semantics or passive constructions are embedded (b), while *wollen* constructions serve to embed action predicates (a).

- *Am Donnerstag* *wollen die Mitglieder der Opec+ in einer Telefonkonferenz mögliche Schritte* *diskutieren.* Die Welt, 07.04.2020 [On Thursday, the members of Opec+ want to discuss possible steps in a conference call.]
- *Selbstständige und Kleinunternehmer* *sollen direkte Zuschüsse von bis zu 15 000 Euro* *bekommen*. Stuttgarter Zeitung, 24.03.2020 [The self-employed and small business owners are to receive direct grants of up to 15,000 euros.]

Teleological *können* formulations always have a potential or circumstantial connotation. In document (c) the formulation of goals is formulated as a possibility from the point of view of the agent of the embedded proposition (here: ›politics‹). In document (d), the target formulation is implemented with an intrasubjective, circumstantial connotation.

c) *Welche* *Rolle kann die Politik in Zukunft* *spielen?* Bild plus, 14.05.2020 [What role can politics play in the future?]
d) *Wir* *können eine ökologische Agrarwende* *angehen [..].* Die Zeit, 14.05.2020 [We can tackle an ecological agricultural turnaround.]

Smirnova and Diewald (2013) discuss origo shifts in reported discourse with present subjunctive and *sollen* constructions. They call constructions with shifted origo »quotative« and constructions without origo shift »reportive« (Smirnova and Diewald 2013, p. 4). They point out that discourse reporting with *sollen* is reportive, i.e. comes without origo shift. In our case, teleological background introduced with *können* are quotative implying an origo shift towards the agent of the objective, while *wollen* and *sollen* constructions are reportive, i.e. the constructed situation is perspectivated from the reporting author’s point of view.

Agents in the formulation of goals are citizens, companies, institutions and states. In the passive *sollen* constructions, the beneficiaries or objects are linguistically constructed as the subjects of the goal formulation.

**CITIZEN**

WILL

NP_[Activity]_ *machen* [do NP_[Activity]_]

NP_[Containing object:bar/institution/shop/restaurant]_ *(wieder) öffnen* [*(re)open* NP_[Containing object:bar/institution/shop/restaurant]_]

*mit* NP_[Counteragent:person in administration/politics]_ *sprechen* [*speak to* NP_[Counteragent:person in administration/politics]_]

KANN

NP_[Activity]_ *machen* [do NP_[Activity]_]

*aus* NP_[EVENT]_ *lernen* [*learn from* NP_[Event]_]

**COMPANY / INSTITUTION**

KANN / WILL

NP_[Action]_ *(wieder) beginnen/starten* [*(re)start* NP_[Action]_]

NP_[Goal]_ *erreichen* [*reach* NP_[Goal]_]

SOLL

*geholfen werden* [*being helped*]

**STATE**

WILL

NP_[Container portal:borders]_ (wieder) *öffnen* [(re)open NP_[Container portal:borders]_]

NP_[Goal]_ *erreichen* [*reach* NP_[Goal]_]

NP_[Benificiary]_ *unterstützen* [*support* NP_[Benificiary]_]

NP_[Money]_ *zahlen* [*pay* NP_[Money]_]

NP_[Benficiary:associations/companies/other state/people]_ *helfen* [*help* NP_[Benficiary:associations/companies/other state/people]_]

**ACTION / REGULATION / RULE**

SOLL

*weiterhin gelten* [be valid further on]

*erfolgen* [take place]

NP_[Process:company failure/virus spread]_ *verhindern* [impede NP_[Process:company failure/virus spread]_]

*beraten werden* [be discussed]

*zu* NP_[Action]_ *dienen* [serve to NP_[Action]_]

NP/CLAUSE_[Action/State]_ *sicherstellen* [ensure NP/CLAUSE_[Action/State]_]

**EVENT / INSTITUTION / TRAFFIC**

SOLL

PP_[State:geschlossen/gesichert/verboten]_ *bleiben* [stay PP_[State:closed/secured/prohibited]_]

##### Epistemic and potential background

**Fig. 12:**
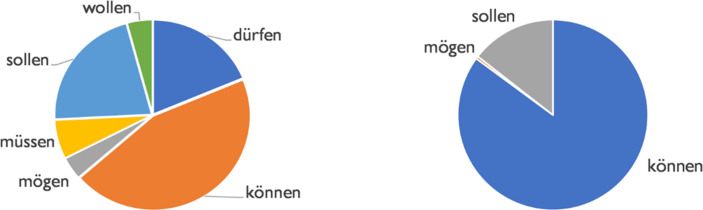
Modals and non-knowledge: epistemic background (left) and potential background (right)

The epistemic and the potential conversational background are treated together here, since the corresponding constructions in all cases presuppose possibilities and thus non-knowledge (Janich 2018). The difference is that the epistemic conversational background involves a subjective evaluation and thus disclosure of personal uncertainty, whereas the potential background constitutes an objective possibility, i.e. suggests an objectivist, rational approach to non-knowledge (Müller/Bartsch/Zinn in press). In addition, it must be considered here that the goal formulations formed with *können* also – as shown above – have epistemic or potential secondary meanings and must therefore be considered when assessing the significance of non-knowledge in the discourse. The epistemic conversational background can be indexed by all modal verbs. The interpretation is often (but not only) triggered by subjunctive forms of verbs and cotextual markers such as epistemic adverbs. The choice of modal verb triggers certain epistemic nuances of meaning in each case, which cannot be discussed here for reasons of space. In our sample (Fig. 12), *können* (45% mostly in the subjunctive: *könnte(n)*) is found above all (a), along with *sollen* (*sollte(n)*; 22%) and *dürfen* (*dürfte(n)*; 19%).

The potential background is usually marked with *können* (in the indicative) (85%). One can see from citation (a) that the possibility indicated here is introduced as conditionally factual with the *können*-construction. The citation is taken from a caption in the context of the interview with a virologist. It is typical in that the construction is often found in reported or quoted expert statements. There are also cases where *sollen* takes over this marking. This is the case in conditional clauses without subjunction, especially when a normative background is indicated in the matrix clause. With *sollen*, the author introduces a factual possibility that is constructed as a constituted condition of the demand in the matrix clause (b). While the potential background is mainly found in statements that revolve directly around COVID-19 in a virologic or medical context, the epistemic background, i.e. the subjective marking of uncertainty, is mainly encountered when talking about social, economic and cultural consequences of the pandemic (c).

- *Die tückischen Coronaviren verbreiten sich per Tröpfcheninfektion: Gelangen sie an Schleimhäute,* *kann man* *sich infizieren.* Bild plus, 04.04.2020 [The treacherous coronaviruses spread by droplet infection: if they get on mucous membranes, you can become infected.]
- *Sollten Bürger in den vergangenen 14 Tagen* *Kontakt zu einem an Covid-19-Erkrankten* *gehabt haben, müssen sie umgehend das Gesundheitsamt […] informieren*. Stuttgarter Zeitung, 04.04.2020 [If citizens have had contact with a person infected with Covid 19 in the past 14 days, they must immediately inform the public health department […].]
- *Die Zahl der infizierten Schlachtarbeiter in Deutschland lag schon vergangenen Freitag bei über 600 und* *dürfte inzwischen* *vierstellig sein.* 14.05.2020 [The number of infected slaughter workers in Germany was already over 600 last Friday and is now likely to be in four figures.]

The patterns of marking epistemic and potential background in the sample show that the topics in the corresponding modal constructions have a wide semantic range. Potential backgrounds can be related to propositions whose topics are medical treatments, citizens, institutions or states. Epistemic backgrounds are invoked when certain developments and data are interpreted and when the consequences and the course of the pandemic are written about from a lay perspective or in the context of interpreting expert statements.

**ACTION / MEDICATION / TREATMENT**

KANN_(potential)_

*helfen* [help]

**CITIZEN**

KANN_(potential)_

*sich mit* NP_[Virus]_ *anstecken* [contract NP_[Virus]_]

**INSTITUTION**

KANN_(potential)_

NP_[Decision]_ *entscheiden* [decide NP_[Decision]_]

**PHENOMENON**

DÜRFTE/KÖNNTE_(epistemic)_

NP_[Meaning]_ / CLAUSE_[Proposition]_ *bedeuten* [mean NP_[Meaning]_ / CLAUSE_[Proposition]_]

*sich um* NP_[Topic:person/__*Spitze des Eisbergs*__]_ *handeln* [be NP_[Topic:person/__*tip of the iceberg*__]_]

**STATE**

DÜRFTE/KÖNNTE_(epistemic)_ / KANN_(potential)_

*(noch lange/eine Weile) dauern* [take long / a while]

**PROCESS**

DÜRFTE/KÖNNTE_(epistemic)_

NP_[Process]_ *folgen* [come on the heels of NP_[Process]_]

*zu* NP_[Process]_ *führen* [result in NP_[Process]_]

KANN_(potential)_

*zu* NP_[Process]_ *führen* [result in NP_[Process]_]

*passieren* [happen]

**VIRUS**

DÜRFTE/KÖNNTE_(epistemic)_

NP_[PATIENT]_ *infizieren* [infect NP_[PATIENT]_]

KANN_(potential)_

*sich ausbreiten* [spread]

NP_[PATIENT]_ *infizieren* [infect NP_[PATIENT]_]

*es* DÜRFTE/KÖNNTE_(epistemic)_ *zu* NP_[Process]_ *kommen* [NP_[Process]_ may occur]

*es* KANN_(potential)_ NP_[Process]_ *geben* [there can be NP_[Process]_]

It is instructive here to look at the distribution of conversational backgrounds across the newspapers in the corpus: The potential background, which indicates rational handling of non-knowledge and specialist discourse, is documented more than four times as often in DIE ZEIT compared to, for example, BILD am Sonntag.^14^ In the case of the epistemic speech background, the distribution is not quite as clear. However, the newspapers that publish discussion articles dominate.^15^

## Summary and discussion

We have seen that modal verbs (other than *wollen* and *mögen*) are significantly overrepresented in press coverage of the first phase of German coverage of COVID-19. The evaluation of a sample of 2,287 occurrences of modal verbs has shown that most modal verb constructions, about one third, point to the normative conversational background. Normative backgrounds are evoked, on the one hand, to address official rules and their effects and, on the other hand in appeals and demands, to refer to social norms that are assumed as common ground. Restrictive circumstances indicated in discourse are mostly related to the life of the individual citizens, while enabling circumstances are related to individual situations, but as well to events and institutions, when discussing perspectives of return to normality (Müller/Zinn 2020). In this context, also teleological backgrounds are introduced. The fact that they account for almost one fifth of the modal verbs in the sample studied highlights the importance of the normalisation perspective in this early phase of the COVID-19 discourse. The epistemic background, on the other hand, does not play as great a role as might have been assumed in a situation characterised by great uncertainty. When the medical and virologic effects of the virus are discussed, potential backgrounds are introduced in assertions of conditional factuality, while epistemic backgrounds are more likely to be found in speculations about the social consequences of the virus. Negated modal constructions are not discourse-constitutive. Restrictions on action tend to be formulated positively. This often happens with hybrid constructions that invoke circumstances and norms in equal measure. Uncertainty, limitations and non-knowledge are neither hidden in discourse nor grammatically mirrored and reinforced, but rather translated into positive formulations of goals. As shown above in chapter 2, this corresponds to the requirements of effective crisis communication, which in this respect can be described as successful. It is particularly noteworthy that the corpus studied here does not capture any top-down one-way communication, but ultimately captures a large piece of the diversity of the polyphonic discourse formations of the media republic via the major national and supra-regional online and print media. So, at least through the keyhole of modal verb analysis, we see a strong uniformity of crisis communication across the various groups of actors, oriented towards the seriousness of the crisis situation. This may be one reason for the general impression that crisis communication in Germany worked comparatively well in the interaction between stately actors, the media and the population in the examined phase March to June 2020. The media largely refrained from criticising the crisis management and supported the line of the governments at the federal and regional levels (Quandt et al. 2020). The politicians in charge largely followed the experts’ advice for action and sought to close ranks, which manifested itself in regular press conferences between the Minister of Health and the head of the Robert Koch Institute, the state institution responsible for the management of infectious diseases and pandemics. And indeed, the measured satisfaction of the population with the government and the pandemic management was high, the measures had very high acceptance (Die ZEIT 2020).

On the other hand, the uniformity that can be grasped here using the example of modal verbs may also be a reason why a counter-movement consisting of radical critics of the measures taken and deniers of the existence of a medical crisis formed very early and very vociferously in Germany, as compared to other countries. This group of people evidentially no longer saw their perspectives represented in the face of the uniformity of crisis communication in the media. These counter-discourses, in turn, are formed primarily in the social media and are reinforced by their communication logics. These could not be captured here on the basis of the available data. In this respect, it must be said that the present analysis only covers one side of the coin. On the other hand, the results reported here may be an important explicative tool for studies dealing with COVID-19 discourses in social media. It remains to add that the discourse formations described here changed fundamentally in the further course of crisis communication in Germany. By spring 2021 at the latest, with massive problems in the vaccination campaign and the need for ever new restrictions, crisis communication itself has turned into a profound communication crisis. This opens up an urgent need for research on many levels. The further development of modal verb constructions is only one, albeit extremely revealing, aspect of this.

## Footnotes

1 Due to space limitations, I provide here only my English translation of the passages. German *müssen* has always been translated with *must* to be clear about the word identity in German. The original speech can be found under the following address: https://www.bundeskanzlerin.de/bkin-de/aktuelles/fernsehansprache-von-bundeskanzlerin-angela-merkel-1732134 [last access 12.03.2021].

2 http://www.rhetorik.uni-tuebingen.de/portfolio/rede-des-jahres/ [last access 27.05.2021].

3 Duden-Grammatik (Wöllstein 2016, p. 468). Breindl (2018) describes these verbs as »core inventory« of the German modal verb system, to be distinguished from »peripheral« constructions such as *brauchen zu* [need to] or *haben zu* [have to] and »semi-modals« such as *scheinen zu* [seem to] oder *pflegen zu* [use to].

4 My translation from German: »Unter einem (einzelnen) REDEHINTERGRUND verstehen wir die Voraussetzungen, im Hinblick auf die der anstehende Sachverhaltsentwurf (als möglich oder notwendig) zu bewerten ist.«.

5 In the literature, there are various proposals as to the level at which alethic modality is to be located. Hundt (2003) proposes a basic distinction between epistemic and alethic modality. He then derives further non-epistemic modal verbs from this. In Dietrich’s (1992) older model, the alethic modality is set as one of five basic relations. The others are the deontic, dispositional, fatalistic/accidental and preferential relation. He interprets epistemic modality as a »relativization« that can be added to the other readings (Dietrich 1992, p. 51).

6 The corpus is part of a series of international corpora on the news coverage on COVID-19. These corpora have been compiled within our research platform www.discourselab.de in cooperation with colleagues at the universities of Heidelberg (Katharina Jacob) and Melbourne (Jens Zinn). We thank Daniel Wachter and Marcel Kückelhaus for their support in corpus compilation and preprocessing.

7 POS-tagging (part-of-speech tagging) is the automatic assignment of part-of-speech categories to words in the corpus.

8 https://spacy.io/.

9 CQL Query – negated modal constructions (example): »([lemma=″sollen″&pos=″VM.*″][word!=″,|:|;″]*[word=″niemand|nicht|weder|kein.*″%c])|([word=″niemand|nicht|weder|kein.*″%c][word!=″,|:|;″]*[lemma=″sollen″&pos=″VM.*″]) within s«.

10 This comparison must not be over-interpreted because the Log-likelihood ratio also depends, among other things, on the absolute frequency of a datum in the two populations measured. Nevertheless, this finding is a clear indication of the great importance of *können* in the corpus.

11 Main verbs in modal verb constructions in query results categorised as normative; f ≥ 3.

12 Ruppenhofer et al. (2016), https://framenet.icsi.berkeley.edu/fndrupal/frameIndex [last access 27.05.2021]. The FrameNet terminology is noted in subscript brackets. If the FrameNet terminology can be specified by non-relational semantic categories collected inductively from the corpus, these are noted in the same bracket after the colon., e.g. NP_[Message:allowance]_.

13 The patterns were searched for by finding the full verbs in each of the categorised documents and manually extracting the propositions embedded in the modal constructions from the verb tokens occurring more than three times. This was done with the following search query in CQL: MU (meet [pos=″VV.+″][pos=″VM.+″] s).

14 Distribution of modals indicating the potential background in the sample (f/1 million words): Die ZEIT (inklusive ZEIT Magazin) 2,001.97*,* Die Welt 1,504.44, Bild plus 1,224.19, FAZ, 1,201.82, Stuttgarter Zeitung 1,184.55, Der Spiegel 1,012.33, taz 856.94, SPIEGEL ONLINE 843.54, BILD am Sonntag, 429.89.

15 Distribution of modals indicating the epistemic background in the sample (f/1 million words): Frankfurter Allgemeine Zeitung 1,764.37, Der Spiegel 1,687.22, Die ZEIT (inklusive ZEIT Magazin) 1,653.80, Bild plus 1,506.70, SPIEGEL ONLINE 1,450.89, Die Welt 1,128.33, Stuttgarter Zeitung 1,114.87, taz, die tageszeitung 1,020.16, BILD am Sonntag 859.78.
